# An Approximate Estimation Approach of Fault Size for Spalled Ball Bearing in Induction Motor by Tracking Multiple Vibration Frequencies in Current

**DOI:** 10.3390/s20061631

**Published:** 2020-03-14

**Authors:** Chidong Qiu, Xinbo Wu, Changqing Xu, Xiang Qiu, Zhengyu Xue

**Affiliations:** College of Marine Electrical Engineering, Dalian Maritime University, Dalian 116026, China; wuxinbo@dlmu.edu.cn (X.W.); xcq2015@dlmu.edu.cn (C.X.); qiuxiang1130@dlmu.edu.cn (X.Q.); xuezy@dlmu.edu.cn (Z.X.)

**Keywords:** induction motor, ball bearing, fault size estimation, dynamic model, friction torque, highlighting fault features, squared envelope spectrum, Hall sensors

## Abstract

Fault size estimation is of great importance to bearing performance degradation assessment and life prediction. Until now, fault size estimation has generally been based on acoustic emission signals or vibration signals; an approach based on current signals has not yet been mentioned. In the present research, an approximate estimation approach based on current is introduced. The proposed approach is easy to implement for existing inverter-driven induction motors without complicated calculations and additional sensors, immune to external disturbances, and suitable for harsh conditions. Firstly, a feature transmission route from spall, to Hertzian forces, and then to friction torque is simulated based on a spall model and dynamic model of the bearing. Based on simulated results, the relation between spall size and the multiple characteristic vibration frequencies in friction torque is revealed. Secondly, the multiple characteristic vibration frequencies modulated in the current is investigated. Analysis results show that those frequencies modulated in the current are independent of each other, without spectrum overlap. Thirdly, to address the issue of which fault features modulated in the current are very weak, a fault-feature-highlighting approach based on reduced voltage frequency ratio is proposed. Finally, experimental tests were conducted. The obtained results validate that the proposed approach is feasible and effective for spall size estimation.

## 1. Introduction

Induction motors play an important role in many industrial and on-board applications because of their low cost, simple construction, and high reliability. It is well known that faulty bearings contribute to most of the failures in rotating machinery [1,2]. Depending on the type and size of the machine, bearing failure distributions vary from about 40% to about 90% from large to small machines [3]. In fact, bearings, even when properly designed, are sensitive components and failure is often due to inadequate operating conditions or failures in maintenance, which are more serious on marine ships than on land, such as excessive loading, shaft misalignment, wrong mounting, improper lubrication, etc. [4].

Fatigue in rolling element bearings, resulting in spalling of the races and rolling elements, is the most common cause of bearing failures. There are three types of fatigue in bearings: surface distress, fatigue pitting and fatigue spalling [5]. Surface distress appears as a smooth surface resulting from plastic deformation in the asperity region (typically less than 10 μm). Pitting appears as shallow craters in contact surfaces with a depth of, at most, the thickness of the work-hardened layer (approximately 10 μm). Spalling leaves deeper cavities at contact surfaces with a depth of 20–100 μm [6].

Bearing faults are generally slowly progressive [7]. The appearance of the first spall does not cause immediate breakdown [8], and also does not mean the end of the bearing’s useful life [9]. A premature removal of the bearing from service may be very expensive [6]. The size of the spalling area has a significant influence on the operation performance and the remaining useful life of rolling element bearings. Therefore, spall size estimation is of great importance to bearing performance degradation assessment and life prediction [9]. If the spall size can be promptly tracked, it is greatly helpful for ensuring correct maintenance decisions and preventing unexpected downtime [10]. 

For the moment, the spall size estimation approach is generally based on acoustic emission signal [2,9,11], or vibration signal [6,10,12,13,14]. Kang et al. [2] introduced a 2-D visualization tool that represents the percentage of the Gaussian-mixture-model-based residual component-to-defect component ratios via time-varying and multi-resolution envelope analysis, then distinguished the different crack sizes based on the k-NN classifier, but the specific fault size was not estimated. Ming et al. [9] used an averaged dual-impulse interval determining method to evaluate the spall size by calculating the autocorrelation function of the squared envelope. Al-Ghamd et al. [11] used the burst duration in acoustic emission signals to estimate the spall size. Sawalhi et al. [6] used cepstrum analysis to give an average estimate of the spacing between the entry and impact events. Zhao et al. [10] used the approximate entropy method and empirical mode decomposition to extract the times of the entry and exit events. Cui et al. [12] used the vertical–horizontal synchronized root mean square to distinguish different fault sizes. Ahmadi et al. [13,14] estimated the defect size by measuring the distance between the entry and impact events. Most of the above approaches implement fault size estimation by determining the interval between two events caused by the same spall.

A common drawback of the above approaches is that they are not suitable for applications in some special and harsh environments which have external vibration or noise interference. In these cases, current signals are preferable as they are immune to external environmental interference. However, the two events caused by the spall are modulated in the alternating current, and are scarcely possible to distinguished in the time domain using the above approaches. Therefore, spall size estimation based on current has not been mentioned until now.

A novel idea which tracks the two events in the frequency domain is proposed in the present work. To address this issue, the feature transmission route from spall, to Hertzian forces, to friction torque, and then to current, is investigated detailedly. Then, the relation between the spall size and the sidebands modulated in current is revealed. However, identifying those sidebands modulated in current is consistently very tricky.

The complex signal transmission route from bearing defect to stator current poses higher impedance and hence results in lower signal-to-noise ratio (SNR) [15]. The amplitude of the torque ripple is very small for real defects; the coefficient between the torque ripple and the current modulation is a damping coefficient [3]. The modulation index is largely less than one [16]. Owing to the poor SNR, the detection and diagnosis of bearings faults by using current signal is still challenging [7].

Furthermore, the current of a typical induction motor involves dominant components such as the supply fundamental and its multiple harmonics, the eccentricity, slot, and saturation harmonics, etc. [17]. For the defect signatures, the dominant components in current are not related to bearing defects, which are usually considered as noise. The suppression of strong noise is then another problem to be solved.

Therefore, highlighting fault features (enhancing SNR) or noise suppression are always a research focus, whether in current or in other signals. Noise suppression in current-based bearing fault diagnosis is focused on elimination of the fundamental supply frequency and its harmonics. To address this problem, some effective approaches are proposed based on notch filters [18], the noise cancellation method using time shifting [17], and the Teager-Kaiser energy operator [16], etc. However, as the power supply frequency is the carrier of defect signatures, excessive noise suppression is not always the most reasonable option. Instead, a simple and effective approach of noise suppression is the squared envelope of signal based on Hilbert transform. The squared envelope of signal retains all the necessary diagnostic information [16], and partly suppresses the power supply frequency and its harmonics. Till now, the squared envelope of signal has been applied successfully in preprocessing of current and other signals [3,17,19,20,21]. 

Focusing on the poor SNR, some effective approaches to highlighting fault features in current have been proposed based on space vector [3], Park’s vector [22], etc. Unlike the above conventional strategies, this paper attempts to highlight fault features based on motor operation under reduced voltage frequency ratio.

Finally, a simple and feasible spall size estimation approach by tracking multiple vibration frequencies in current is introduced. The proposed approach only needs to calculate the squared envelope spectrum, an algorithm which is very simple, mature and widely used in various fields. Therefore, the proposed approach is easy to implement for existing inverter-driven induction motors without complicated calculation and additional sensors, immune to external disturbances, and suitable for harsh conditions.

Two major contributions have been made in this work. First, the relation between the spall size and the sidebands modulated in the current is revealed based on the simulation results for a spall model and a dynamic model of bearings; then, a novel idea which estimates the spall size by tracking the current sidebands is introduced. Second, this work introduces a fault-feature-highlighting approach based on reduced voltage frequency ratio, which is more effective than the traditional approaches.

This work is organized as follows. In Section 2, the fluctuation of friction torque caused by spall, the relation between spall size and multiple characteristic vibration frequencies, and the multiple characteristic vibration frequencies modulated in current, are investigated. In Section 3, the fluctuation of friction torque is verified based on a customized measuring instrument, and the spall size estimation approach by tracking multiple vibration frequencies is verified based on experimental platform. Finally, the conclusions and remarks are given in Section 4.

## 2. Theoretical Analysis

### 2.1. Elastic Deformation

For ball bearings, widely employed in induction motors, elastic deformation between raceways and balls produces a non-linear phenomenon between force and deformation, which is obtained by the Hertzian theory [23]. The non-linear relation of load-deformation is given by

(1) $$F=K\delta^{1.5}$$

where *F* is Hertzian contact force, *K* is the load-deflection factor or constant for Hertzian contact elastic deformation, *δ* is the radial deflection or contact deformation.

The load–deflection factor *K* depends on the contact geometry. Total deflection between two raceways is the sum of the approaches between the rolling elements and each raceway. *K* is given by [24].

(2) $$K={\left[1/\left({\left(1/K_{i}\right)}^{2/3}+{\left(1/K_{o}\right)}^{2/3}\right)\right]}^{3/2}$$

where *K_i_* is inner-raceway-to-ball contact stiffness, and *K_o_* is outer-raceway-to-ball contact stiffness. 

The detailed formulas and parameters for *K_i_* and *K_o_* are introduced in the literature [23,24,25]. The value of *K* for an SKF 6206 bearing is 4.9582 × 107 N/m, according with to the value range proposed in [25,26,27].

### 2.2. Spall Model of Bearings

**Figure 1:**
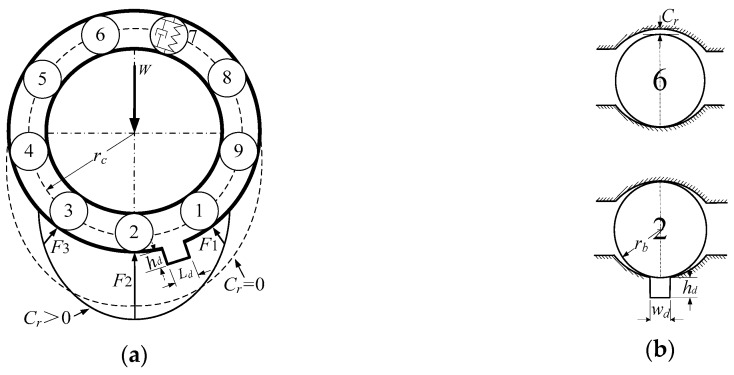
Spall in outer raceway: (**a**) Front view of bearing with spall; (**b**) View along with ball rolling direction.

We considered the spall in the outer raceway as a rectangular notch of depth *h_d_* with a width of *w_d_* and a length of *L_d_* shown in Figure 1. Take into account that the spall depth is generally larger than the radial deflection of outer raceways, and the spall width is also larger than the diameter of the contact deformation area between raceways and balls. Therefore, the spall size estimation in the present research focused on different spall lengths, *L_d_*.

The rolling element–raceway contact can be considered as a spring mass system, in which the outer race is fixed in a rigid support and the inner race is fixed rigidly with the motor shaft. In Figure 1a, the 7th ball is shown as the symbol of spring mass, the other balls are only shown as a number for convenience.

Generally, ball bearings are designed with clearance *C_r_* for flexible and unblocked operation [23]. On account of the effect of clearance *C_r_*, the number of balls providing rotor support varies. If *C_r_* = 0, there are five balls (4, 3, 2, 1, 9) providing rotor support as shown in Figure 1a. If *C_r_* > 0, there are three balls (3, 2, 1) providing rotor support. For an SKF 6206 bearing, the clearance *C_r_* is generally about 5–20 μm. Therefore, the balls in the area between 5 o’clock and 7 o’clock principally provide rotor support; it is clear that the effect on the motor is most severe for the spall located in this support area.

Because the load–deflection factor *K* is very large, the contact deformation *δ* is correspondingly very small, and generally micrometer-scale. The contact deformation area between the ball and the race is very small; the rise and fall of contact force will be obvious even for a very small spall. When the 2nd ball rolls into the spall, the contact force *F_2_* of the 2nd ball disappears, the centre of the inner race connected with the motor shaft falls, the contact force *F_1_* and *F_3_* subsequently rises, and the vibration is also produced at this moment.

**Figure 2:**
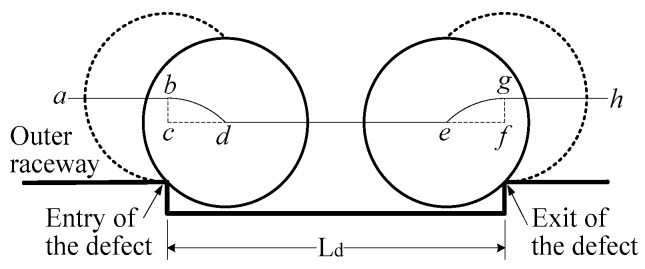
The rolling path of centre of the ball from entry to the exit of the spall.

For further exploration of the effect of spall, a detailed rolling path is shown in Figure 2.

Considering the balls as point masses is a common approach employed in numerous literatures [24,28,29,30], in which the contact force of the ball entirely disappears as soon as the centre of the ball enters into the entry of the spall. When the ball passes through the spall from the entry to the exit of the spall, the common rolling path of the centre of the ball is considered to be in a sequence of “*a*-*b*-*c*-*d*-*e*-*f*-*g*-*h*” as shown in Figure 2. In reality, considering the size of the ball [25,31,32,33,34], the rolling path of the centre of the ball is in a sequence of “*a*-*b*-*d*-*e*-*g*-*h*”. Till the “*d*” point, the contact force of the ball just entirely disappears; beginning at the “*e*” point, the contact force of the ball already arises. 

**Figure 3:**
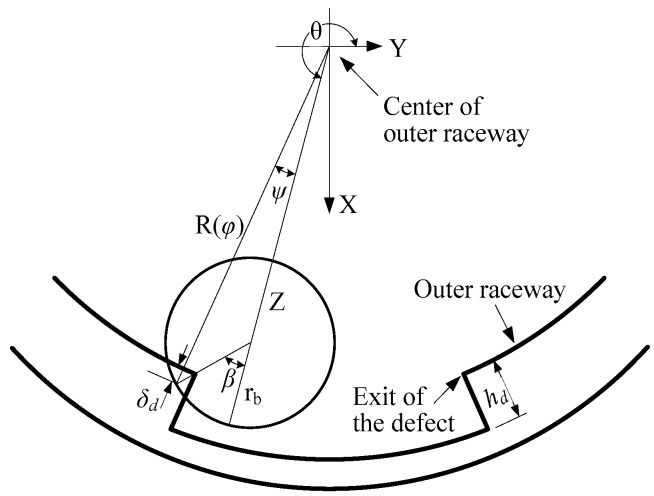
Schematic diagram of the ball passing the spall in the outer raceway.

In order to estimate accurately the effect of spall, the present research adopted the spall model introduced by Alireza Moazen Ahmadi [35], which reasonably considers the size of the ball. The schematic diagram of the ball passing the spall in the outer raceway is as shown in Figure 3.

The spall in the outer raceway can be modeled as

(3) $$\gamma \left(\theta \right)=\left\{ \begin{array}{l} h_{d},\begin{array}{cc} & \theta_{en}<\theta < \end{array}\theta_{ex}\\ 0,\begin{array}{cc} & \mathrm{otherwise} \end{array} \end{array}\right.$$

where *θ* is the angular position of the ball, *θ_en_* and *θ_ex_* are the angular positions of the spall entry and exit, and *h_d_* is the spall depth. The geometry function of the outer raceway is given by

(4) $$R\left(\varphi \right)=\left(r_{c}+r_{b}\right)+C_{r}+\gamma \left(\varphi \right)$$

where *r_c_* is bearing pitch radius, *r_b_* is the radius of the ball, and *C_r_* is the radial clearance of the bearing.

The contact deformation *δ* is given by

(5) $$\delta \left(\beta \right)=\frac{Z+r_{b}\cos\beta }{\cos\psi }-R\left(\theta +\psi \right)$$

where *Z* is the distance of the ball from the centre of the outer raceway, *β* is the angle between the maximum deformation point on the ball and Z based on the centre of the ball, and *ψ* is the angle between the maximum deformation point on the ball and Z based on the centre of the outer raceway. The *ψ* is given by

(6) $$\psi =\tan^{-1}\left(\frac{r_{b}\sin\beta }{Z+r_{b}\cos\beta }\right)$$

The contact deformation *δ_d_* between the entry and the exit of the spall is finally determined by

(7) $$\delta_{d}=\max\left[\delta \left(\beta \right)\right];\begin{array}{cc} & -\frac{\pi }{2} \end{array}<\beta <\frac{\pi }{2}$$

### 2.3. Dynamic Model of Bearings

**Figure 4:**
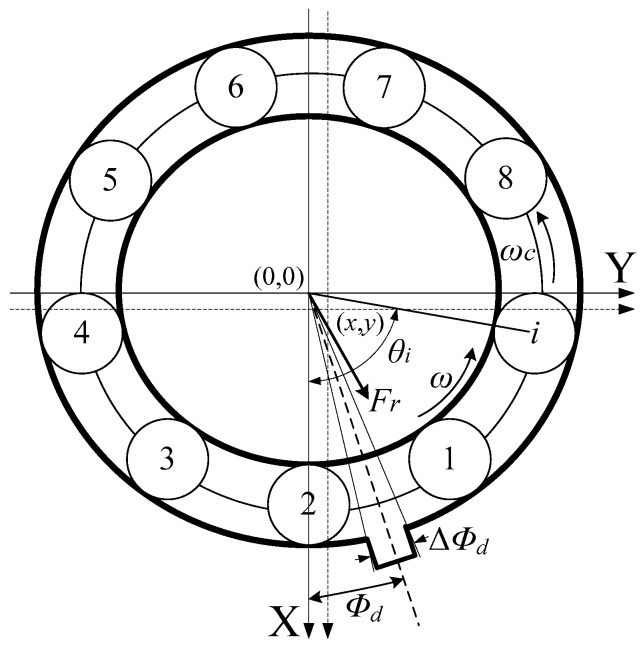
Dynamic model of bearing.

Because the ball bearing primarily supports the radial forces, the dynamic model based on a two-degrees-of-freedom system is enough for analysing the defect feature transmission route from spall, to Hertzian forces, and then to friction torque. The two-degrees-of-freedom system is also employed widely in the majority of literature up to now [24,25,28,29,30,31,32]. The dynamic model of bearings is shown in Figure 4. *Φ_d_* is the angle from the X-axis to the centre of the spall, and Δ*Φ_d_* is the angle corresponding to the spall length *L_d_*. For convenience, Δ*Φ_d_* is directly regarded as the spall length in the following part. 

If *x* and *y* are the deflections along the X-axis and the Y-axis, the radial deflection *δ_i_* of the *i*^th^ ball at any angle *θ_i_* is given by

(8) $$\delta_{i}=\left\{ \begin{array}{l} \delta_{d}\begin{array}{cc} & \Phi_{d}-\Delta \Phi_{d}/2<\theta_{i}<\Phi_{d}+\Delta \Phi_{d}/2 \end{array}\\ \left(x\cos\theta_{i}+y\sin\theta_{i}\right)-C_{r}\begin{array}{cc} & \mathrm{otherwise} \end{array} \end{array}\right.$$

The *Z* in Equation (5) is given by

(9) $$Z=r_{c}+\left(x\cos\theta_{i}+y\sin\theta_{i}\right)$$

Based on Equation (1), resolving the total Hertzian forces along the X-axis and Y-axis:

(10) $$\begin{array}{l} F_{X}=\sum_{i=1}^{N}K{\left(\delta_{i}\right)}_{+}^{1.5}\cos\theta_{i}\\ F_{Y}=\sum_{i=1}^{N}K{\left(\delta_{i}\right)}_{+}^{1.5}\sin\theta_{i} \end{array}$$

where *N* is the number of balls. Since the Hertzian contact force arises only when there is contact between the ball and the raceway, the respective contact force is set to zero when the contact deformation is equal or smaller than zero. This is indicated by subscript “+”.

*θ_i_* is given by

(11) $$\theta_{i}=\omega_{c}t+\frac{2\pi }{N}\left(i-1\right),\begin{array}{cc} & i=1,\ldots ,N. \end{array}$$

where *ω_c_* is the speed of the cage.

(12) $$\omega_{c}=\frac{1}{2}\left[\omega \left(1-\frac{D_{b}}{D_{c}}\right)\right]$$

where *ω* is the shaft speed.

Based on the structural dynamics theory, the equations of motion for a two-degrees-of-freedom system can be written as follows:

(13) $$\begin{array}{l} m\ddot{x}+c\dot{x}+F_{X}=W\\ m\ddot{y}+c\dot{y}+F_{Y}=0 \end{array}$$

where *m* is the mass of the rotor and of the inner race, *c* is the damping factor, and *W* is the static load. The damping factor *c* is commonly chosen to be 200 Ns/m [24,25,27,28,29].

### 2.4. Load Dependent Friction Torque

A reasonable estimate of the friction torque of a given rolling bearing under moderate load and speed conditions is the sum of load friction torque *M*_1_ and viscous friction torque *M_v_*. *M_v_* is primarily dependent on speed and the method of lubrication [23] and is independent of bearing load. Hence, *M_v_* isn’t going to be discussed because of its insensitivity to bearing defects in this paper.

The load dependent friction torque is given by

(14) $$M_{1}=f_{1}F_{\beta }D_{c}$$

where *f*_1_ is a factor dependent on the bearing design, and *F_β_* depends on the magnitude and direction of the applied load. The detailed formulas and parameters for *f*_1_ are introduced by literature [23].

For ball bearings having a nominal contact angle 0°, there is not applied axial load, so *F_β_* equals the applied radial load *F_r_*. For static equilibrium, the applied radial load must equal the sum of the Hertzian forces from each of the balls.

### 2.5. Simulated Hertzian Forces and Friction Torque

The spall model and dynamic model of the bearing were solved in MATLAB using the ordinary differential equation solver (ode45). For the defective SKF 6206 bearing in a 2.2 kW induction motor, the parameters of the spall model and dynamic model of the bearing are given in Table 1.

In Table 1, the number of balls *N*, ball radius *r_b_*, pitch radius *r_c_*, radial clearance *C_r_* and load-deflection factor *K* are parameters related to the SKF 6206 bearing. The defect depth *h_d_*, angle of the defect centre *Φ_d_* and angle of the defect length Δ*Φ_d_* are parameters related to the assumed spall in bearing outer raceway. The mass of the rotor *m* and static load *W* are parameters related to the 2.2 kW induction motor, and the *W* is equal to *m* times gravity acceleration. For the bearing SKF 6206 mounted in the motor, the damping factor *c* is commonly chosen to be 200 Ns/m. The motor speed *n* and shaft speed *ω* are parameters related to the motor operation, and *ω* = 2πn/60.

**Figure 5:**
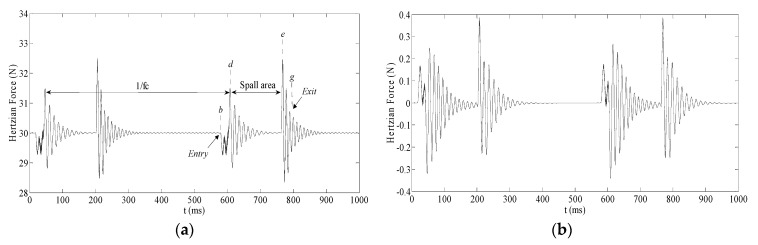
The total Hertzian forces along the X-axis and Y-axis: (**a**) X-axis total Hertzian forces *F_X_*; (**b**) Y-axis total Hertzian forces *F_Y_*.

The total Hertzian forces along the X-axis and Y-axis are as shown in Figure 5.

In Figure 5, because *Φ_d_* is 0°, which means that the spall is just located in the 6 o’clock region, the X-axis total Hertzian forces *F_X_* is much larger than the *F_Y_*, which will be ignored in following discussion.

Whether in *F_X_* or in *F_Y_*, the cycle of the Hertzian forces caused by the spall exactly corresponds to the characteristic vibration frequencies *f_c_*, which are given by:

(15) $$f_{c}=0.5Nf_{r}\left[1-\left(D_{b}\cos\alpha \right)/D_{c}\right]$$

where *f_r_* is the mechanical rotor frequency, *N* is the number of balls, *D_b_* is the diameter of the ball, *D_c_* is bearing pitch diameter, and *α* is the contact angle between the ball and the raceway.

Except for *f_c_*, the information about spall size is also revealed distinctly in Figure 5. There are two impulse responses, respectively located at the entry and exit of the spall. The spall size corresponds to the primary interval between “d” and “e”, the additional interval between “b” and “d”, and the additional interval between “e” and “g”. The primary interval is easy to determine based on Figure 5. The latter two additional intervals are not very distinct, but can be estimated by size of the ball. 

Through different pathways, two impulse responses caused by the entry and exit of the spall can also be propagated to the vibration signal and the acoustic emission signal. Then, spall size estimation can be implemented by determining the average interval between two events [2,6,9,10,11,12,13,14].

**Figure 6:**
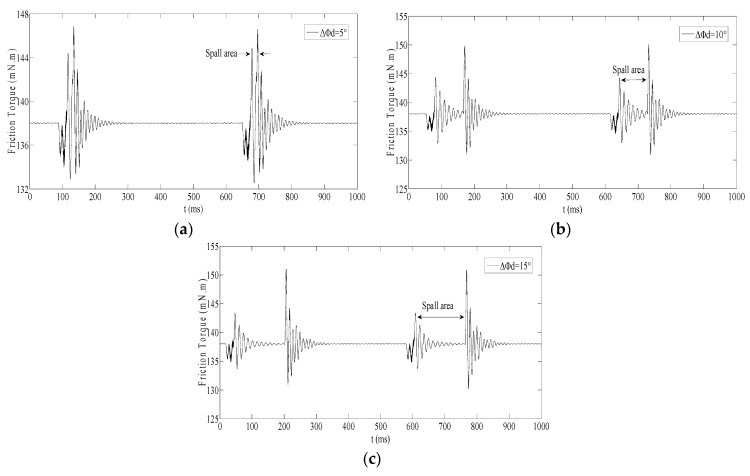
The friction torque for different spall length: (**a**) Δ*Φ_d_*=5°; (**b**) Δ*Φ_d_*=10°; (**c**) Δ*Φ_d_*=15°.

Whereafter, the Δ*Φ_d_* is set as 5°, 10° and 15° respectively in Table 1; the friction torques are as shown in Figure 6.

In Figure 6, the two impulse responses caused by the two events exhibit consistent in friction torque for different spall lengths, but the amplitude of the friction torque is very small. For spall size estimation based on current, not only the friction torque will be buried, likely in load torque, but also the friction torque modulated in current will be faced with strong noise which will include the power supply frequency and its harmonic. All this means that spall size estimation based on current is scarcely possible by determining the average interval in the time series. As an alternative, in the frequency domain, tracking the frequency feature of the friction torque modulated in current will be a feasible solution.

Unlike a single-point defect, which only produces a single characteristic vibration frequency, the spall including two impulse responses will produce multiple characteristic vibration frequencies. Therefore, the relation between spall length and the multiple characteristic vibration frequencies in friction torque must be investigated first.

### 2.6. The Relation Between Spall Size and Multiple Characteristic Vibration Frequencies

For constant spall depth *h_d_* = 100 μm, Changing the Δ*Φ_d_* from 1° to 20° with a distance of 1°, the friction torque was respectively solved. For each friction torque, the amplitudes of 1st~3rd multiple characteristic vibration frequencies *f_c_*, 2*f_c_* and 3*f_c_* were achieved based on Fast Fourier Transform (FFT) in MATLAB. Only the *f_c_*, 2*f_c_* and 3*f_c_* frequencies are analyzed in this paper because of the sufficient spall length information carried by these three frequencies, even though other, higher, multiple-characteristic vibration frequencies are also present. 

**Figure 7:**
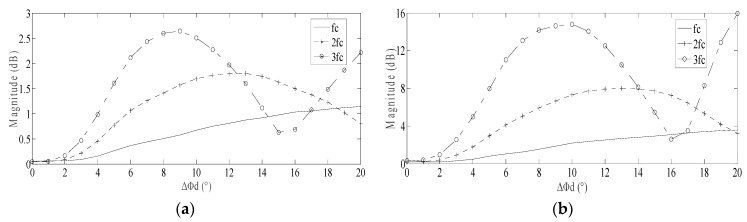

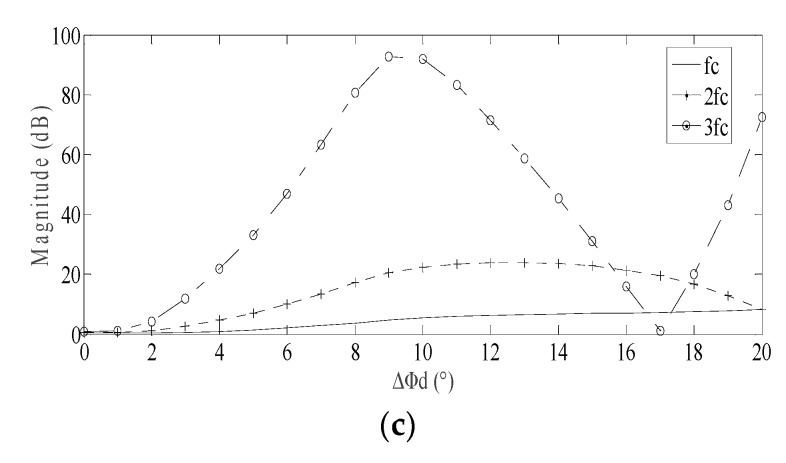
Amplitude trend of *f_c_*, 2*f_c_* and 3*f_c_* for different spall length when the motor speed is varied respectively: (**a**) Motor speed *n* = 150 r/min; (**b**) Motor speed *n* = 300 r/min; (**c**) Motor speed *n* = 450 r/min.

When the motor speed was set as 150, 300 and 450 r/min respectively, the amplitude trend of *f_c_*, 2*f_c_* and 3*f_c_* in friction torque for various spall length were as shown in Figure 7.

In Figure 7, following the increase of the spall length, the fluctuation of amplitude of 2*f_c_* and 3*f_c_* are more obvious than *f_c_*. When Δ*Φ_d_* reached a certain level (10° when motor speed is 300r/min shown in Figure 7b), the amplitude of 3*f_c_* reached its maximum. When the Δ*Φ_d_* further increased (15°–16°), the amplitude of 3*f_c_* reduced to the minimum; in the meantime, the amplitude of 2*f_c_* reached its maximum. The regular rise and fall of amplitude of 2*f_c_* and 3*f_c_* was consistent for different motor speeds. When the motor speed is slower, the amplitude trend collectively shifts left, as shown in Figure 7a. When the motor speed is higher, the amplitude trend collectively shifts right, as shown in Figure 7c.

Following an increase in the motor speed, the amplitudes of *f_c_*, 2*f_c_* and 3*f_c_* all increase. Theoretically, increased motor speed is possibly beneficial for identifying those spall-related characteristic frequencies in stator current.

**Figure 8:**
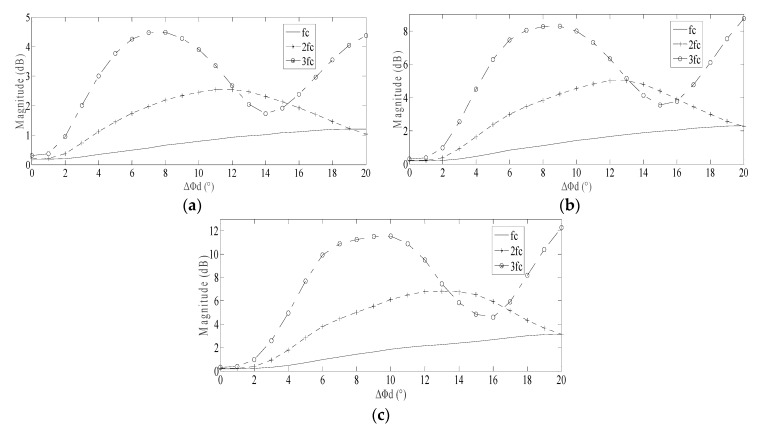
Amplitude trends of *f_c_*, 2*f_c_* and 3*f_c_* for different spall lengths when the spall depth is varied respectively: (**a**) Spall depth *h_d_* = 20 μm; (**b**) Spall depth *h_d_* = 40 μm; (**c**) Spall depth *h_d_* = 60 μm.

In order to verify that the regular rise and fall of amplitude of 2*f_c_* and 3*f_c_* is universal for other cases, the various spall depth is also discussed. When the motor speed was set as 300 r/min, for three spall depths *h_d_* = 20, 40 and 60 μm respectively, the amplitude trends of *f_c_*, 2*f_c_* and 3*f_c_* were as shown in Figure 8.

In Figure 8, for different spall depths, the regular rise and fall of amplitude of 2*f_c_* and 3*f_c_* is still subsistent. In addition, following an increase in the spall depth, the amplitudes of *f_c_*, 2*f_c_* and 3*f_c_* just increase a little. That is to say, the amplitude of the multiple characteristic vibration frequencies is not very sensitive for spall depth.

Based on termly observation of current during the motor operation, the spall length may be estimated approximately by tracking the regular rise and fall of amplitude of 2*f_c_* and 3*f_c_*. The estimation of spall length will be sufficient evidence for the correct maintenance decision and a reasonable maintenance schedule for the induction motor.

### 2.7. The Multiple Characteristic Vibration Frequencies Modulated in Current

The friction torque is transmitted to the stator current by means of nonlinear phase modulation, which, even though a single frequency, can also be evolved into a chain of frequencies in current. If there are spectrum overlaps among the multiple characteristic vibration frequencies modulated in the current, spall size estimation based on the regular rise and fall of amplitude of 2*f_c_* and 3*f_c_* will be imprecise. Therefore, the multiple characteristic vibration frequencies modulated in current will be investigated.

Only taking account of the effect of *f_c_*, 2*f_c_* and 3*f_c_*, which are the primary parts of fluctuant friction torque caused by spall, the total load torque *Τ_l_* can be described as follows:

(16) $$T_{l}(t)=T_{0}+\sum_{n=1}^{3}T_{n}\cos(n\omega_{c}t)$$

where *Τ_0_* is the constant torque, *n* is a positive integer, *ω_c_* is the characteristic vibration angular frequency and *Τ_n_* is the amplitude of *nω_c_*.

Based on the same method introduced in [36], the stator current is given by

(17) $$i(t)=I_{1}\sin\left(\omega_{s}t+\sum_{n=1}^{3}\beta_{n}\cos(n\omega_{c}t)\right)$$

where *ω_s_* is the electrical supply angular frequency, *β_n_* is the modulation index of the *n*^th^ multiple characteristic vibration angular frequency *nω_c_*, and *β_n_* is given by

(18) $$\beta_{n}=\frac{p{Τ}_{n}}{J{\left(n\omega_{c}\right)}^{2}}$$

where *p* is the pole pairs and *J* is the total inertia of the rotating system. Following an increase of *n*, *β_n_* will decrease. 

After modulating in current, the amplitude of 2*f_c_* will be less than that shown in Figure 7; the amplitude of 3*f_c_* will also be lesser, but the regular rise and fall is unaltered. For this reason, those other higher order multiple characteristic vibration frequencies are not taken into account, because those modulated in current are scarcely possible to identify.

Equation (17) can be expanded on the basis of the first-kind Bessel function

(19) i(t)=I1Im[ejωstej∑n=13βncos(nωct)]=I1Im[ejωst∏n=13∑m=−∞∞Jm(βn)ejmnωct]

where the symbol “Im” represents taking the imaginary part, *m* is an integer, and *J_m_*(*β_n_*) is the *m*^th^ Bessel function for *β_n_*.

For *n*=1, 2, and 3, Equation (19) can be simplified as

(20) i(t)=I1Im[ejωst∑a=−∞∞Ja(β1)ejaωct∑b=−∞∞Jb(β2)ejb2ωct∑c=−∞∞Jc(β3)ejc3ωct]=I1Im[∑a,b,c=−∞∞Ja(β1)Jb(β2)Jc(β3)ej(ωst+aωct+b2ωct+c3ωct)]=I1∑a,b,c=−∞∞Ja(β1)Jb(β2)Jc(β3)sin[ωst+(a+2b+3c)ωct]

where *a*, *b*, and *c* are integers, and similar to *m* in (19). *J_a_*(*β*_1_) is the *a*^th^ Bessel function for *β*_1_, *J_b_*(*β*_2_) is the *b*^th^ Bessel function for *β*_2_, and *J_c_*(*β*_3_) is the *c*^th^ Bessel function for *β*_3_.

Letting *k* = *a* + 2*b* + 3*c*, then, the bearing fault-related frequency *f_bf_* in stator current is given by

(21) $$f_{bf}=|f_{s}\pm kf_{c}|$$

where *f_s_* is the power supply frequency; *f_s_* ± *kf_c_* is commonly called the *k*^th^ sideband in phase modulation. 

Equation (21) is same as the proposed result in the majority of literature, but the difference is that the amplitude of side frequencies in current can be revealed using Equation (20).

For the general formula of *J_a_*(*β*_1_), *J_b_*(*β*_2_), and *J_c_*(*β*_3_), *J_m_*(*β_n_*) can be expressed as

(22) $$J_{m}(\beta_{n})=\sum_{k=0}^{\infty }{\left(-1\right)}^{k}\frac{\beta_{n}^{m+2k}}{2^{m+2k}k!\Gamma (m+k+1)}(m\geq 0)$$

Considering that

(23) Γ(m+k+1)=(m+k)!

equation (22) can be rewritten as

(24) $$J_{m}(\beta_{n})=\sum_{k=0}^{\infty }{\left(-1\right)}^{k}\frac{\beta_{n}^{m+2k}}{2^{m+2k}k!(m+k)!}(m\geq 0)$$

Considering that the magnitude of *β_n_* is extremely small (less than 0.1) [3,16,36], only *k* = 0 deserves attention. This therefore leads to

(25) $$J_{m}(\beta_{n})\approx \frac{\beta_{n}^{m}}{2^{m}m!}(m\geq 0)$$

Equation (25) can be directly rewritten as

(26) $$J_{m}(\beta_{n})\approx \left\{ \begin{array}{ll} 1 & m=0\\ 0.5\beta_{n} & m=1\\ 0 & m\geq 2 \end{array}\right.$$

In addition, the symmetry of the Bessel function is shown, as *J_-m_*(*β_n_*) = −*J_m_*(*β_n_*) when *m* is odd, and *J_-m_*(*β_n_*) = *J_m_*(*β_n_*) when *m* is even. 

Focusing on the amplitude of the first sideband *f_s_*+*f_c_*, *k*=1, that is, *a*+2*b*+3*c*=1. There are innumerable solutions for *a*, *b*, and *c*. 

Based on Equation (26) and the symmetry of the Bessel function, only 0, 1 or −1 is a significant value for *a*, *b*, and *c*. For *a* + 2*b* + 3*c* = 1, there are three significant solutions: *a* = 1, *b* = 0, *c* = 0; *a* = −1, *b* = 1, *c* = 0; or *a* = 0, *b* = −1, *c* = 1. These solutions are put into Equation (20), then, the amplitude of *f_s_*+*f_c_* is presented as *I*_1_*J*_1_(*β*_1_)*J*_0_(*β*_2_)*J*_0_(*β*_3_) −*I*_1_*J*_1_(*β*_1_)*J*_1_(*β*_2_)*J*_0_(*β*_3_) −*I*_1_*J*_0_(*β*_1_)*J*_1_(*β*_2_)*J*_1_(*β*_3_). Based on Equation (26), the amplitude of *f_s_* + *f_c_* can be further simplified as 0.5*I*_1_*β*_1_−0.25*I*_1_*β*_1_*β*_2_−0.25*I*_1_*β*_2_*β*_3_.

Considering that *β_n_* is extremely small, the magnitudes of 0.25*I*_1_*β*_1_*β*_2_ and 0.25*I*_1_*β*_2_*β*_3_ are much smaller than the magnitude of 0.5*I*_1_*β*_1,_ and are ignorable. The amplitude of *f_s_* + *f_c_* is close to 0.5*I*_1_*β*_1_.

In the same way, the amplitude of other sidebands can be calculated. The final conclusion is that the amplitude of *f_s_* ± *f_c_* is 0.5*I*_1_*β*_1_; the amplitude of *f_s_* ± 2*f_c_* is 0.5*I*_1_*β*_2_; the amplitude of *f_s_* ± 3*f_c_* is 0.5*I*_1_*β*_3_. 

This conclusion means that, after transmitting to current, the spall size information implied in the 1st~3rd multiple characteristic vibration frequencies presents perfectly in the current without spectrum overlap. In brief, the amplitude of *f_s_* ± 2*f_c_* is overwhelmingly contributed by the 2nd multiple characteristic vibration frequencies, 2*f_c_*, and is unaffected by the other multiple characteristic vibration frequencies. The condition of *f_s_* ± *f_c_* and *f_s_* ± 3*f_c_* is similar to that of *f_s_* ± 2*f_c_*. 

In theory, spall size estimation based on current can be implemented by tracking the regular rise and fall of amplitude of multiple characteristic vibration frequencies modulated in current.

## 3. Experimental Verification

### 3.1. Experimental Verification on Fluctuant Friction Torque

Based on the simulation results in Section 2.5, there are two impulse responses respectively located at the entry and exit of the spall, which present in friction torque. To verify this simulation result, experimental verification is necessary. However, in practice, the friction torque of a spalled bearing assembled in a motor, is hard to be measured directly. Therefore, an indirect experimental scheme is implemented for only the spalled bearing instead of the bearing assembled in motor. Theoretically, because there are differences in just two parameters, mass of the rotor and static load, the amplitudes of two impulse responses will be weakened overall, but the forms of two impulse responses stay the same. 

**Figure 9:**
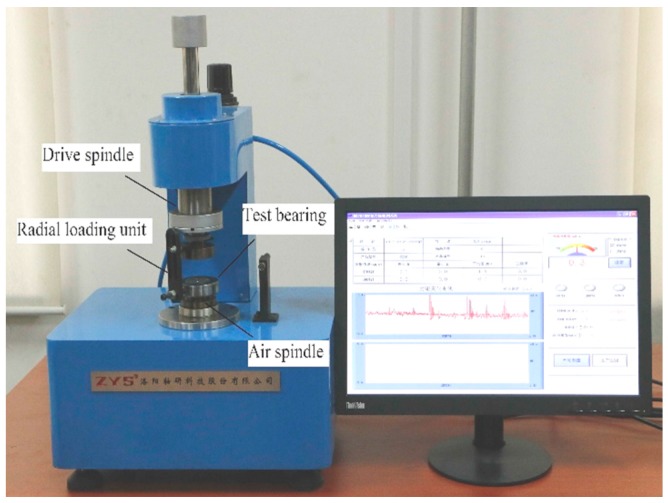
M9908B bearing friction torque measuring instrument.

The M9908B bearing friction torque measuring instrument produced by Luoyang Bearing Science & Technology Co. Ltd in China, was used in the present research. The M9908B is as shown in Figure 9.

The technical specifications of the M9908B are as follows: torque range is 50 mN.m; rotational speed of drive spindle is 5 rpm; error is ± 0.5 mN.m; repeatability is ± 0.5 mN.m.

Like the majority of bearing friction torque measuring instruments (for example Vibrac BRG 3000), an axial load is commonly used in the testing of bearings with M9908B. It is clear that the fluctuation of friction torque caused by spall cannot be perfectly shown by means of axial loading. Hence, a customized radial loading unit was supplemented to M9908B.

The bearing’s outer ring is driven by the additional radial loading unit fixed in the drive spindle. The bearing’s inner ring is fixed in the air spindle, connected with a torque transducer. When the bearing’s outer ring rotates along with the drive spindle, because of the presence of the friction torque, the bearing’s inner ring shows a tendency of rotation along with the outer ring. The rotation tendency of the inner ring will be impeded by a torque transducer; for dynamic equilibrium, the friction torque was measured.

**Figure 10:**
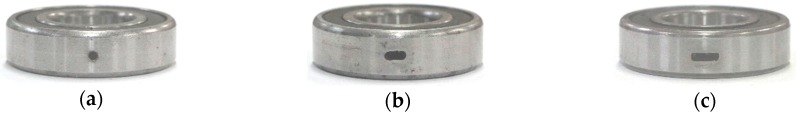
Bearings with different spall length: (a) Δ*Φ_d_*=5°; (b) Δ*Φ_d_*=10°; (c) Δ*Φ_d_*=15°.

Basing on electro-discharge machining, the outer raceway of the SKF 6206 bearings was artificially drilled with an approximate angle of spall length of 5°, 10° and 15° respectively, as shown in Figure 10.

**Figure 11:**
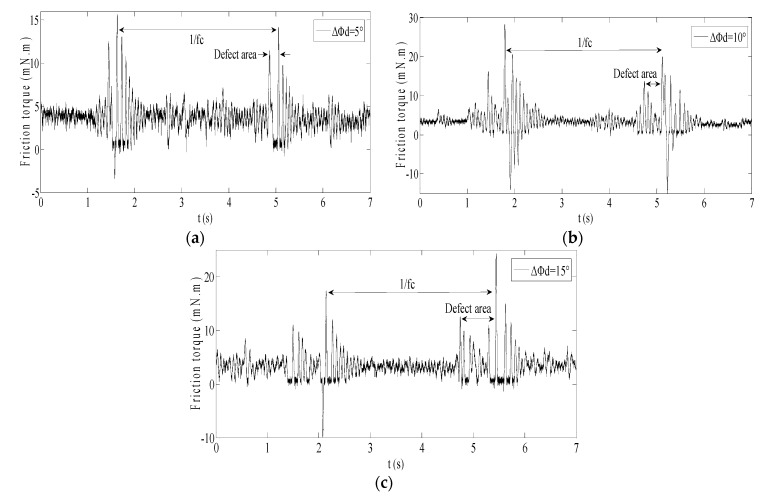
Friction torque of bearing with different spall length: (**a**) Δ*Φ_d_* = 5°; (**b**) Δ*Φ_d_* = 10°; (**c**) Δ*Φ_d_* = 15°.

For the above three defective bearings, the friction torques achieved respectively based on M9908B were as shown in Figure 11.

As Figure 11 shows, the simulation results in Section 2.5 were verified experimentally:

1. The cycle of the fluctuant friction torque exactly corresponded to the characteristic vibration frequencies *f_c_*.
2. There were two impulse responses when the ball passed through the spall.
3. The interval between the two impulse responses approximatively corresponded to the spall length.

Therefore, the regular rise and fall of amplitude of 2*f_c_* and 3*f_c_* in friction torque caused by the interval between the two impulse responses is feasible for estimating spall length.

### 3.2. Experimental Verification on Stator Current

#### 3.2.1. Experimental Platform

**Figure 12:**
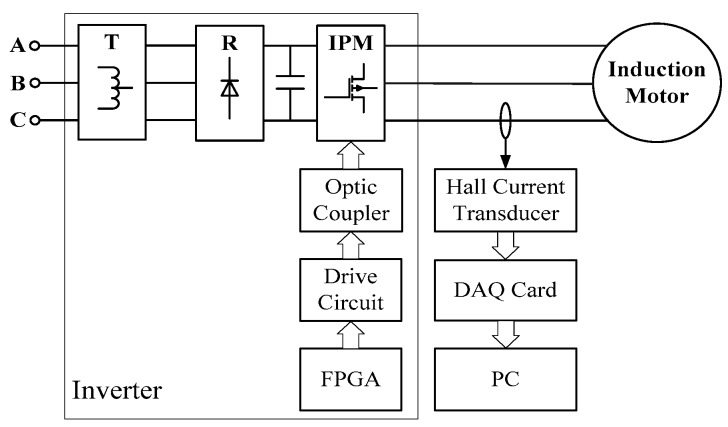
Schematic diagram of a drive system based on FPGA.

An experimental platform was designed based on FPGA and IPM. The schematic diagram is shown as Figure 12.

In Figure 12, T is a 3-phase autotransformer; R is a 3-phase rectifier; IPM is a Mitsubishi Intelligent Power Modules PM50CSD120, with a collector current of 50 A and collector-emitter voltage of 1200V; the Optic coupler is a HCPL 4504; the drive circuit is a triode 9013; FPGA is an Altera Cyclone IV E devices EP4CE15F17C8. SPWM modulation mode was employed in the FPGA drive system operating at carrier frequency 20 kHz.

The parameters of three-phase induction motor were as follows: rated power 2.2 kW; rated voltage 380 V; rated frequency 50 Hz; rated current 5.03 A; rated speed 1430 r/min; pole pairs 2; bearing type SKF 6206. 

Induction motor speed was measured by handheld tachometer. Induction motor stator current was measured by Hall current sensors LEM IT60-S, whose measuring range was 60A. Linearity error was 20 ppm, and frequency bandwidth was 500 kHz. 

**Figure 13:**
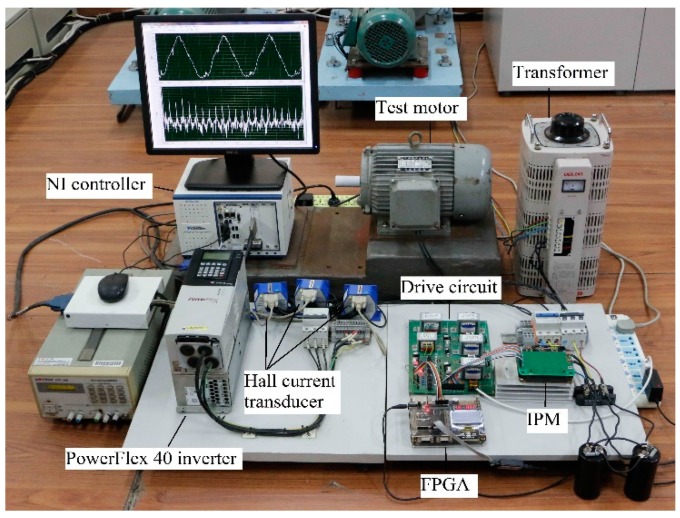
The picture of experimental platform.

The current data was sampled by National Instruments X series multifunction data acquisition card PXIe-6356, with an analog digital converter resolution of 16 bits, sampling rate of 1.25 MS/s, and bandwidth of 1 MHz. PC is a National Instruments 2.3 GHz quad-core embedded controller PXIe-8135. A picture of the experimental platform is shown as Figure 13.

Theoretically, the lower the motor speed is, the more distinct the two impulse responses in friction torque are, without overlap between two impulse responses. However, in practice, when the power supply frequency is set too low, the fault-related frequencies probably overlap with power supply frequency and its harmonic, because of the wide lobe produced by spectrum analysis. Therefore, the power supply frequency is initially set to 10 Hz.

In order to avoid false positive detection caused by the fluctuation of mechanical load or shaft coupling, the motor was in no-load. 

To ensure reasonable comparison among spectrum analysis results, the Welch method was adopted for spectrum analysis throughout the following research. The specific parameters were as follows: FFT points were 8192, the sampling points were 8192, sampling frequency was 1000 Hz.

#### 3.2.2. Highlighting Fault Features Based on Reduced Voltage Frequency Ratio

For tracking multiple characteristic vibration frequencies modulated in current, the squared envelope of current based on Hilbert transform is employed to suppress noise. However, this method alone is far from enough; therefore, this paper attempts to improve the SNR based on additional approaches.

To supply enough electromagnetic torque to the load, the motor commonly operates in rated main flux, in which the voltage frequency ratio is always constant in common inverter. Because the amplitude of friction torque is much less than the amplitude of electromagnetic torque, the fluctuant friction torque is filtered rapidly by motor inertia before modulating in current, which dynamic process is very transient. Accordingly, the fault features modulated in current are very weak.

Under the constant power supply frequency without any form of closed-loop control, the main flux and the electromagnetic torque of the motor will be reduced following the reduction of supply voltage; meanwhile, the fluctuant friction torque caused by spall is almost unchanged. When the supply voltage drops to a certain extent, the electromagnetic torque is also close to the friction torque. In which case, when the ball passes through the spall, the fault features will be preferably modulated in the current due to the longish dynamic interactive process between the fluctuant friction torque and the electromagnetic torque, together with the slower motor speed. Consequently, the fault features modulated in the current will be highlighted.

**Figure 14:**
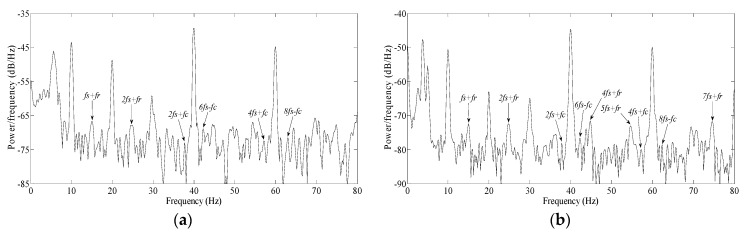

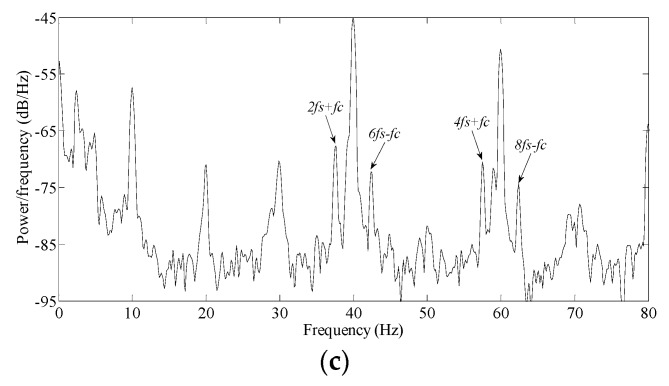
The squared envelope spectrum of stator current: (**a**) voltage frequency ratio 76 V/10 Hz; (**b**) voltage frequency ratio 38 V/10 Hz; (**c**) voltage frequency ratio 19 V/10 Hz.

In the experimental platform in Figure 13, the power supply frequency was held constant at 10 Hz, the motor was in no-load, and the bearing had a spall length of 5° (Figure 10a). For the experimental motor, normal voltage frequency ratio was 380 V/50 Hz (7.6). The voltage frequency ratio was reduced gradually for verifying the effect of changed main flux based on the FPGA and IPM drive system. During experiment, the voltage frequency ratio was set as 76 V/10 Hz (7.6), 38 V/10 Hz (3.8), 19 V/10 Hz (1.9) respectively; the measured motor speed was 299 r/min, 298 r/min, 293 r/min, and the *f_c_* was 17.7 Hz, 17.7 Hz, 17.4 Hz accordingly. The squared envelope spectra of the stator currents in all of above cases were as shown as Figure 14. 

In Figure 14a, when the voltage frequency ratio was normal (7.6), there were four fault frequencies: 2*f_s_* + *f_c_*, 6*f_s_* − *f_c_*, 4*f_s_* + *f_c_*, 8*f_s_* − *f_c_*. All these frequencies were below the average of spectral lines, and therefore are not suitable for use as a diagnosis index. A similar situation is also presented in Figure 14b, where the voltage frequency ratio was half of normal (3.8).

Following the reduction of voltage frequency ratio further, the motor entered the critical state between rotation and non-rotation. For the experimental motor, when the voltage frequency ratio was a quarter of normal (1.9), the motor just held a steady rotation speed. In Figure 14c, the four fault frequencies: 2*f_s_* + *f_c_*, 6*f_s_* − *f_c_*, 4*f_s_* + *f_c_*, 8*f_s_* − *f_c_* are unusually noticeable. Consequently, the objective of enhancing the SNR of defect signatures is achieved perfectly.

**Figure 15:**
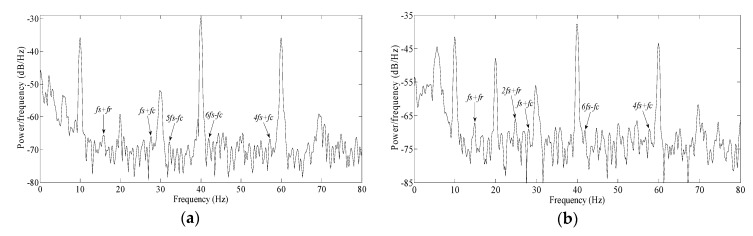
The squared envelope spectra based on traditional approaches when normal voltage frequency ratio is normal: (**a**) instantaneous symmetrical components; (**b**) Park’s vector.

In order to verify the performance of proposed method, some traditional approaches were also used to compare. The instantaneous symmetrical components and Park’s vector are often used to highlight fault features based on three phase currents. When the voltage frequency ratio was normal, the squared envelope spectra based on traditional approaches were as shown as Figure 15.

In Figure 15a, there are four fault frequencies: *f_s_* + *f_c_*, 5*f_s_* − *f_c_*, 6*f_s_* − *f_c_* and 4*f_s_* + *f_c_* are identifiable. In Figure 15b, there are three fault frequencies: *f_s_* + *f_c_*, 6*f_s_* − *f_c_* and 4*f_s_* + *f_c_* are identifiable. All these frequencies are more distinct than those in Figure 14a. Therefore, the fault features may be highlighted based on instantaneous symmetrical components and Park’s vector. 

However, the performance of fault feature highlighting based on traditional approaches is far inferior to the proposed method, as is shown in Figure 14c. In fact, because the motor is in no-load, the defect signatures are inappreciable under low bearing radial load, which was always a challenging issue facing low load up to now. Nevertheless, the proposed method addresses this issue by reducing the electromagnetic torque to a certain extent, just matching weak friction torque. In this case, it is inevitable that the fault features will be highlighted perfectly. 

Furthermore, the proposed method may be also applicable to loaded motors without any form of closed-loop control.

#### 3.2.3. The Effect of Rotation Speed on Current Sideband

The amplitudes of characteristic vibration frequencies increase slightly following an increased motor speed, based on the theoretical analysis in Section 2.6. The effect of rotation speed on current sideband will be investigated in the present section.

On one hand, the optimal power supply frequency must be selected. On the other hand, the multiple characteristic vibration frequencies need to be presented collectively for intercomparison. Considering the above two points, the bearing with spall length of 10° (Figure 10b), is employed in experimental motor, the motor is in no-load, and the voltage frequency ratio is set as a quarter of normal (1.9) throughout. 

**Figure 16:**
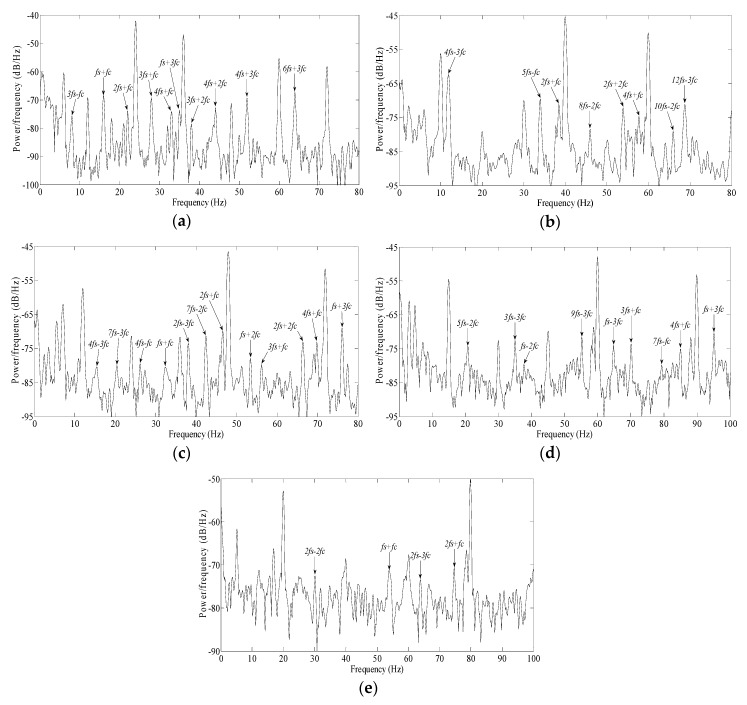
The squared envelope spectrum for different power supply frequency: (**a**) 6 Hz; (**b**) 10 Hz; (**c**) 12 Hz; (**d**) 15 Hz; (**e**) 20 Hz.

During the experiment, the power supply frequencies were set as 6 Hz, 10 Hz, 12 Hz, 15 Hz and 20 Hz respectively; the measured motor speeds were 162 r/min, 290 r/min, 354 r/min, 444 r/min and 589 r/min, and *f_c_* values were 9.6 Hz, 17.2 Hz, 21 Hz, 26.3 Hz and 34.9 Hz accordingly. The squared envelope spectra of stator currents in all of above cases were as shown as Figure 16. 

In Figure 16a, the power supply frequency is 6 Hz, a number of fault frequencies arise, far beyond the average of spectral lines. The sidebands of *3f_c_* included: *6f_s_+3f_c_*, *4f_s_+3f_c_* and *f_s_+3f_c_*. The sidebands of *2f_c_* included: *4f_s_+2f_c_* and *3f_s_+2f_c_*. The sidebands of *f_c_* included: *f_s_*+*f_c_*, *3f_s_+f_c_*, *2f_s_*+*f_c_*, *3f_s_*−*f_c_* and *4f_s_*+*f_c_*. Among all fault frequencies, *6f_s_+3f_c_*, modulated from *3f_c_*, was maximal, next was *f_s_*+*f_c_* and *4f_s_+2f_c_*. Theoretically, this is a suitable power supply frequency for investigating the multiple modulated frequencies. However, the modulated frequencies were too close to each other, and several modulated frequencies even overlapped with the power supply frequency and its harmonics.

In Figure 16b, the power supply frequency was 10Hz, and number of fault frequencies arose similarly. For simplicity, no more enumerating every frequency; only the most prominent frequency among each group frequencies will be chosen for comparison. Obviously, *4f_s_−3f_c_* was maximal, next was *5f_s_−f_c_* and *2f_s_+2f_c_*. Because there was no sideband overlap with the power supply frequency and its harmonics, this is a very suitable power supply frequency for investigating the multiple modulated frequencies.

In Figure 16c, the power supply frequency was 12 Hz. *f_s_+3f_c_* was maximal, next was *2f_s_ + f_c_* and *7f_s_−2f_c_*. On the whole, the amplitudes of all fault frequencies were slightly lower than above two cases. 

In Figure 16d, the power supply frequency was 15 Hz. *f_s_+3f_c_* was maximal, next was *3f_s_+f_c_* and *5f_s_−2f_c_*. For the sidebands of *2f_c_*, except the prominent frequency *5f_s_−2f_c_*, there was only one frequency, *f_s_−2f_c_*, which is almost masked in spectral lines. Some fault frequencies were also not prominent, as in the above three cases.

In Figure 16e, the power supply frequency was 20Hz. *2f_s_+f_c_* was maximal, next was *2f_s_−2f_c_* and *2f_s_−3f_c_*. The majority of fault frequencies fell to the average of the spectral lines, and also were not prominent. 

The amplitudes of characteristic vibration frequencies in friction torque increase slightly following increased motor speed, based on the theoretical analysis in Section 2.6. However, after modulating to current, the extent of increase is negligible based on the above spectrum analysis. 

In fact, from 15 Hz (Figure 16d), the fault frequencies begin to decline. Till 20 Hz (Figure 16e), the fault frequencies are already infrequent and unobvious. The reason is that the ideal motion trail just can be met for lower motor speed. When the motor speed is higher, the sharp collision appears between the ball with raceways. The ideal motion trail of the ball is disturbed by the irregular collision, the feature of multiple characteristic vibration frequencies is also weakened.

Consequently, when the power supply frequency is set below 15 Hz, this is a reasonable option for tracking multiple characteristic vibration frequencies modulated in the current. 

In practice, anywhere below 15 Hz is a feasible observation point, provided that there is no sideband overlap with power supply frequency and its harmonics. 

Accordingly, the power supply frequency was set to 10 Hz in following investigation.

#### 3.2.4. Spall Size Estimation

Based on the conclusion proposed in Section 2.6, a regular rise and fall should be present in the amplitude of multiple characteristic vibration frequencies in friction torque. Corresponding to the power supply frequency, 10 Hz, the motor speed was focused at 300 r/min. In that case, the theoretical analysis result between spall size and multiple characteristic vibration frequencies in friction torque is as shown in Figure 7b.

After the fluctuant friction torque is modulated to current, the amplitudes of multiple characteristic vibration frequencies will be weakened partly. According to the theoretical analysis result in Section 2.7, the modulation index will decrease gradually, followed with an increase of order of characteristic vibration frequencies. That is to say, the amplitudes of sidebands of *3f_c_* and *2f_c_* will be lower than the result shown in Figure 7b; meanwhile, the amplitude of sidebands of *f_c_* will be higher. 

In fact, this is verified by the above investigation. In Figure 16b, the bearing with spall length of 10°, the sideband of *3f_c_* was maximal, but was not very high, like that shown in Figure 7b. Meanwhile, the sideband of *f_c_* was not very low, like that shown in Figure 7b. Similar situations are also present in Figure 16a, Figure 16c, and Figure 16d.

**Figure 17:**
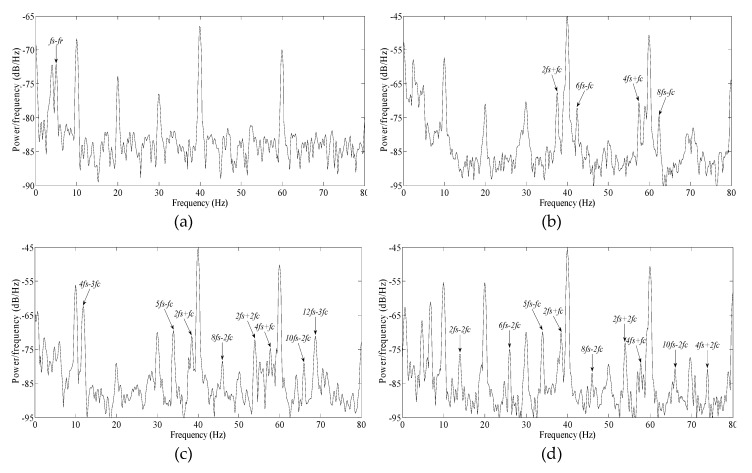
The squared envelope spectra for normal bearings and faulty bearings with various spall lengths: (**a**) normal bearing; (**b**) Δ*Φ_d_*=5°; (**c**) Δ*Φ_d_*=10°; (**d**) Δ*Φ_d_*=15°.

Using the experimental platform in Figure 13, the motor was in no-load, and the voltage frequency ratio was always set as 19V/10Hz during the following experiment. Three SKF 6206 bearings were artificially drilled, with an approximate angle of spall length of 5°, 10° and 15° respectively, as shown in Figure 10. A normal bearing, and the above three defective bearings, were installed in the test motor successively. The measured motor speeds were 294 r/min, 293 r/min, 290 r/min, 289 r/min, and the *f_c_* values were 17.4 Hz, 17.4 Hz, 17.2Hz, 17.1Hz, accordingly. The squared envelope spectra of the stator currents in all of above cases were as shown as Figure 17. 

It should be noted that those frequencies, below power supply frequency, will not be useful as a diagnosis index, because there may be several adjacent frequencies modulated by multiple harmonics present in this area.

In Figure 17a, for a normal bearing, except for *f_s_*−*f_r_*, there is no fault frequency present in spectrum. 

In Figure 17b, for a defective bearing with spall length of 5°, several fault frequencies arise, far beyond the average of spectral lines. The sidebands of *f_c_* include: *2f_s_+f_c_*, *6f_s_−f_c_*, *4f_s_*+*f_c_* and *8f_s_*−*f_c_*. There are not obvious sidebands for 2*f_c_* and 3*f_c_*. One reason is that two impulse responses caused by spall partly overlap each other. Another reason is that the modulation indices of 2*f_c_* and 3*f_c_* are less than that of *f_c_*.

In Figure 17c, for defective bearings with spall lengths of 10°, a number of fault frequencies arose. The sidebands of *3f_c_* include: *4f_s_−3f_c_* and *12f_s_−3f_c_*. The sidebands of *2f_c_* include: *2f_s_+2f_c_* and *10f_s_−2f_c_*. The sidebands of *f_c_* include: 5*f_s_*−*f_c_*, *2f_s_+f_c_* and *4f_s_*+*f_c_*. 

According to the trend shown in Figure 7b, as the amplitudes of *3f_c_* and *2f_c_* increased gradually from 5° to 10°, so, the sidebands of *3f_c_* and *2f_c_* emerged increasingly in the current. Because the increase of *3f_c_* is more obvious than others, *4f_s_−3f_c_* is maximal among all these sidebands; next is *5f_s_−f_c_* and *2f_s_+2f_c_*, which is also generally consistent with the trends shown in Figure 7b, considering the additional effect of the modulation index.

In Figure 17d, for defective bearings with spall length of 15°, there were more fault frequencies present in the spectrum. The sidebands of *2f_c_* include: *2f_s_+2f_c_*, *6f_s_−2f_c_*, *2f_s_−2f_c_*, *10f_s_−2f_c_*, *4f_s_+2f_c_* and *8f_s_−2f_c_*. The sidebands of *f_c_* include: *5f_s_−f_c_*, *2f_s_+f_c_* and *4f_s_*+*f_c_*. 

According to the trend shown in Figure 7b, as amplitudes of *3f_c_* are decreased gradually from 10° to 15°, so the sidebands of *3f_c_* disappeared in the current. As the amplitudes of *2f_c_* increased slightly from 10° to 15°, so, a number of sidebands of *2f_c_* became present in the current. On the other hand, as the amplitudes of *f_c_* always increase slightly from 5° to 15° based on Figure 7b, so, the *5f_s_−f_c_* was maximal among all these sidebands; next is *2f_s_+2f_c_*.

From Figure 17b to Figure 17d, the sidebands of *f_c_* are always present, but the amplitude of sidebands did not increase visibly. For this reason, the spall size isn’t estimated based on the amplitudes of sidebands of *f_c_*.

In general terms, the regular rise and fall of sidebands of 3*f_c_* and *2f_c_* in the current are verified based on Figure 17, which is generally consistent with the trend shown in Figure 7b, considering the additional effect of the modulation index. Consequently, some remarkable results can be summarized:

1. When the spall is a small size (spall length less than 5°, below a 2.5mm spall length for a 6206 bearing), only the sidebands of *f_c_* are visible in the current.
2. When the spall is a little larger (angle of spall length about 6°–10°, approximately a 3–5mm spall length for a 6206 bearing) the sidebands of 2*f_c_* and 3*f_c_* are gradually more obvious. Following the increase of the spall length, the increase of the sidebands of 3*f_c_* becomes more obvious than others; finally, the sidebands of 3*f_c_* reach a maximum.
3. When the spall is large (angle of spall length about 11°–15°, approximately a 5.5–7.5mm spall length for a 6206 bearing), following the increase of the spall length, the sidebands of 3*f_c_* weaken gradually; finally, the sidebands of 3*f_c_* disappear entirely. Meanwhile, the sidebands of 2*f_c_* become more obvious gradually.

Consequently, based on the proposed approaches, the spall size in a bearing’s outer raceway can be estimated approximately by tracking the multiple characteristic vibration frequencies in the current.

When the spall length enlarges further, the vibration and harsh sound caused by the spall are very obvious; therefore, the larger spall length isn’t necessary to investigate.

## 4. Conclusions

This paper proposes an approximate fault size estimation approach based on current, which is immune to external environmental interference, addressing the shortcomings of existing approaches which are not suitable for applications in some special and harsh environments. The present research reveals the relation between the spall size and the sidebands modulated in current, proposes a novel idea to estimate spall size by tracking the current sidebands, and introduces a fault-feature-highlighting approach based on reduced voltage frequency ratio. Some noteworthy conclusions are presented as follows:

1. Compared to traditional fault-feature-highlighting approaches based on instantaneous symmetrical components and Park’s vector, the proposed approach has showed better performance.
2. Below lower power supply frequencies, the proposed fault size estimation approach can effectively track fault size.
3. The proposed fault size estimation approach only needs to calculate the squared envelope spectrum, an algorithm which is very simple, Therefore, this approach is easy to implement for existing inverter-driven induction motors without complicated calculations and additional sensors, and is suitable for harsh conditions.
4. Compared to existing fault size estimation approaches, the drawback of the proposed approach is lower estimation accuracy. Therefore, this approach is only suitable for applications in bearing performance degradation assessment and maintenance decision making for induction motors in service.

## Figures and Tables

**Table 1:** Parameters of Bearing Dynamic Model (based on SKF 6206).

Parameters	Values
Number of balls *N*	9
Ball radius *r_b_*	4.8 × 10^-3^ m
Pitch radius *r_c_*	2.3 × 10^-2^ m
Radial clearance *C_r_*	1.0 × 10^-5^ m
Defect depth *h_d_*	1.0 × 10^-4^ m
Angle of the defect centre *Φ_d_*	0°
Angle of the defect length Δ*Φ_d_*	15°
Shaft speed *ω*	3.14 rad/s
Motor speed *n*	30 r/min
Mass of the rotor *m*	3 kg
Load-deflection factor *K*	4.9582 × 10^7^ N/m
Damping factor *c*	200 Ns/m
Static load *W*	30 N

## References

[1] Tian J., Morillo C., Azarian M.H., Pecht M. (2016). Motor bearing fault detection using spectral kurtosis-based feature extraction coupled with k-nearest neighbor distance analysis. IEEE Trans. Ind. Electron..

[2] Kang M., Kim J., Wills L.M., Kim J.M. (2015). Time-varying and multiresolution envelope analysis and discriminative feature analysis for bearing fault diagnosis. IEEE Trans. Ind. Electron..

[3] Immovilli F., Bellini A., Rubini R., Tassoni C. (2010). Diagnosis of bearing faults in induction machines by vibration or current signals: A critical comparison. IEEE Trans. Ind. Appl..

[4] Harmouche J., Delpha C., Diallo D. (2015). Improved fault diagnosis of ball bearings based on the global spectrum of vibration signals. IEEE Trans. Energy Convers..

[5] Ojaghi M., Sabouri M., Faiz J. (2018). Analytic model for induction motors under localized bearing faults. IEEE Trans. Energy Convers..

[6] Sawalhi N., Randall R.B. (2011). Vibration response of spalled rolling element bearings: Observations, simulations and signal processing techniques to track the spall size. Mech. Syst. Signal Process..

[7] Dalvand F., Kalantar A., Safizadeh M.S. (2016). A novel bearing condition monitoring method in induction motors based on instantaneous frequency of motor voltage. IEEE Trans. Ind. Electron..

[8] Frosini L., Harlisca C., Szabó L. (2015). Induction machine bearing fault detection by means of statistical processing of the stray flux measurement. IEEE Trans. Ind. Electron..

[9] Ming A.B., Zhang W., Qin Z.Y., Chu F.L. (2015). Dual-impulse response model for the acoustic emission produced by a spall and the size evaluation in rolling element bearings. IEEE Trans. Ind. Electron..

[10] Zhao S.F., Liang L., Xu G.H., Wang J., Zhang W.M. (2013). Quantitative diagnosis of a spall-like fault of a rolling element bearing by empirical mode decomposition and the approximate entropy method. Mech. Syst. Signal Process..

[11] Al-Ghamd A.M., Mba D. (2006). A comparative experimental study on the use of acoustic emission and vibration analysis for bearing defect identification and estimation of defect size. Mech. Syst. Signal Process..

[12] Cui L.L., Huang J.F., Zhang F.B. (2017). Quantitative and localization diagnosis of a defective ball bearing based on vertical–horizontal synchronization signal analysis. IEEE Trans. Ind. Electron..

[13] Ahmadi A.M., Howard C.Q. (2016). A defect size estimation method based on operational speed and path of rolling elements in defective bearings. J. Sound Vib..

[14] Ahmadi A.M., Howard C.Q., Petersen D. (2016). The path of rolling elements in defective bearings: Observations, analysis and methods to estimate spall size. J. Sound Vib..

[15] Li D.Z., Wang W., Ismail F. (2015). A spectrum synch technique for induction motor health condition monitoring. IEEE Trans. Energy Convers..

[16] Pineda-Sanchez M., Puche-Panadero R., Riera-Guasp M., Perez-Cruz J., Roger-Folch J., Pons-Llinares J., Climente-Alarcon V., Antonino-Daviu J.A. (2013). Application of the Teager–Kaiser energy operator to the fault diagnosis of induction motors. IEEE Trans. Energy Convers..

[17] Dalvand F., Dalvand S., Sharafi F., Pecht M. (2017). Current noise cancellation for bearing fault diagnosis using time-shifting. IEEE Trans. Ind. Electron..

[18] Immovilli F., Cocconcelli M. (2017). Experimental investigation of shaft radial load effect on bearing fault signatures detection. IEEE Trans. Ind. Appl..

[19] Leite V.C.M.N., Borges Da Silva J.G., Veloso G.F.C., Borges Da Silva L.E., Lambert-Torres G., Bonaldi E.L., De Lacerda De Oliveira L.E. (2015). Detection of localized bearing faults in induction machines by spectral kurtosis and envelope analysis of stator current. IEEE Trans. Ind. Electron..

[20] Elbouchikhi E., Choqueuse V., Amirat Y., Benbouzid M.E.H., Turri S. (2017). An efficient Hilbert-Huang transform-based bearing faults detection in induction machines. IEEE Trans. Energy Convers..

[21] Xu L., Chatterton S., Pennacchi P. (2018). A novel method of frequency band selection for squared envelope analysis for fault diagnosing of rolling element bearings in a locomotive powertrain. Sensors.

[22] Soualhi A., Clerc G., Razik H. (2013). Detection and diagnosis of faults in induction motor using an improved artificial ant clustering technique. IEEE Trans. Ind. Electron..

[23] Harris T.A., Kotzalas M.N. (2006). Essential Concepts of Bearing Technology.

[24] Patil M.S., Mathew J., Rajendrakumar P.K., Desai S. (2010). A theoretical model to predict the effect of localized defect on vibrations associated with ball bearing. Int. J. Mech. Sci..

[25] Kulkarni P.G., Sahasrabudhe A.D. (2014). A dynamic model of ball bearing for simulating localized defects on outer race using cubic hermite spline. J. Mech. Sci. Technol..

[26] Guo Y., Parker R.G. (2012). Stiffness matrix calculation of rolling element bearings using a finite element/contact mechanics model. Mech. Mach. Theory.

[27] Mitsuya Y., Sawai H., Shimizu M., Aono Y. (1998). Damping in vibration transfer through deep-groove ball bearings. J. Tribol..

[28] Harsha S.P., Sandeep K., Prakash R. (2003). The effect of speed of balanced rotor on nonlinear vibrations associated with ball bearings. Int. J. Mech. Sci..

[29] Tiwari M., Gupta K., Prakash O. (2000). Dynamic response of an unbalanced rotor supported on ball bearings. J. Sound Vib..

[30] Gomez J.L., Bourdon A., André H., Rémond D. (2016). Modelling deep groove ball bearing localized defects inducing instantaneous angular speed variations. Tribol. Int..

[31] Sawalhi N., Randall R. (2008). Simulating gear and bearing interactions in the presence of faults: Part I. The combined gear bearing dynamic model and the simulation of localised bearing faults. Mech. Syst. Signal Process..

[32] Sopanen J., Mikkola A. (2003). Dynamic model of a deep-groove ball bearing including localized and distributed defects. Part 1: Theory. Proc. Inst. Mech. Eng. Part K J. Multi-body Dyn..

[33] Petersen D., Howard C., Prime Z. (2015). Varying stiffness and load distributions in defective ball bearings: Analytical formulation and application to defect size estimation. J. Sound Vib..

[34] Niu L.K., Cao H.R., He Z.J., Li Y.M. (2015). A systematic study of ball passing frequencies based on dynamic modeling of rolling ball bearings with localized surface defects. J. Sound Vib..

[35] Ahmadi A.M., Petersen D., Howard C. (2015). A nonlinear dynamic vibration model of defective bearings—The importance of modelling the finite size of rolling elements. Mech. Syst. Signal Process..

[36] Blödt M., Chabert M., Regnier J., Faucher J. (2006). Mechanical load fault detection in induction motors by stator current time-frequency analysis. IEEE Trans. Ind. Appl..

